# The Optimal Exponent Base for emPAI Is 6.5

**DOI:** 10.1371/journal.pone.0032339

**Published:** 2012-03-05

**Authors:** Andrzej Kudlicki

**Affiliations:** Department of Biochemistry and Molecular Biology, Sealy Center for Molecular Medicine, University of Texas Medical Branch, Galveston, Texas, United States of America; Universitat Autònoma de Barcelona, Spain

## Abstract

Exponentially Modified Protein Abundance Index (emPAI) is an established method of estimating protein abundances from peptide counts in a single LC-MS/MS experiment. EmPAI is defined as 10^PAI^ minus one, where PAI (Protein Abundance Index) denotes the ratio of observed to observable peptides. EmPAI was first proposed by Ishihama et al [1] who found that PAI is approximately proportional to the logarithm of absolute protein concentration. I define emPAI65 = 6.5^PAI^-1 and show that it performs significantly better than emPAI, while it is equally easy to compute. The higher accuracy of emPAI65 is demonstrated by analyzing three data sets, including the one used in the original study [1]. I conclude that emPAI65 ought to be used instead of the original emPAI for protein quantitation.

## Introduction

The objective of protein identification studies based on liquid chromatography and mass spectrometry (LC-MS) is to detect the presence of large numbers of proteins in the experimental sample. LC-MS data can be also used to estimate the abundances of particular proteins, and several methods have been developed for this purpose (e.g. [2], [3]), including methods based on spectral counting [1], [3]. The APEX approach [3] relies on estimating the probabilities of observing each peptide from every protein and is therefore difficult to implement; here I discuss the simpler emPAI method of Ishihama et al.

The dependence between the number of detected peptides and absolute concentration of a protein has been demonstrated by [4]. Specifically, the Protein Abundance Index (PAI) has been defined as the ratio of the number of observed peptides to the number of observable peptides.

Ishihama et al. have subsequently observed that PAI is approximately proportional to the logarithm of the protein concentration [1]. Based on this empirical observation, they concluded that the relationship between PAI and molar protein concentration is an exponential function, and proposed to use 10 as the exponent base, noting that the thus defined predictor (emPAI = 10^PAI^-1) provides an acceptable approximation. The formula is phenomenological, but, for its ease of use and availability (e.g. implementation within MASCOT [5] or the standalone EmPAICalc [6]), emPAI has become very popular. However when emPAI was defined, its authors did not report testing whether a better approximation could be obtained by using an exponential function with a base other than 10.

## Analysis

**Figure 1:**
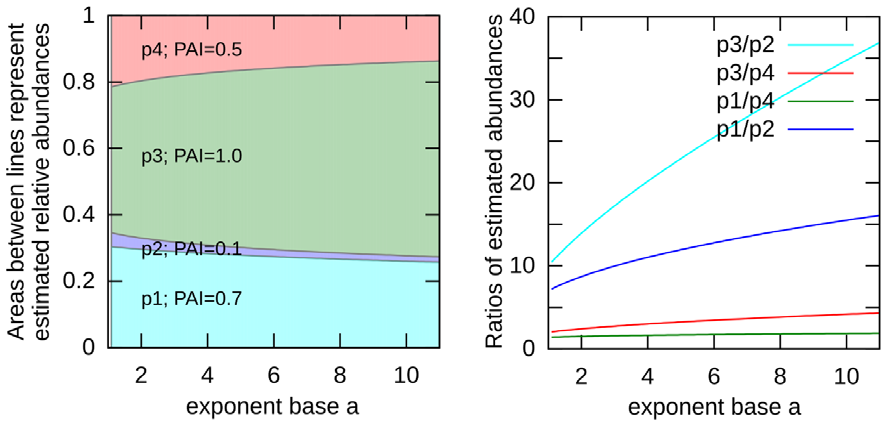
The estimated protein abundances depend on exponent base. Relative protein concentrations estimated using the Generalized Exponentially Modified Protein Abundance Index depend on the base of the exponent. Here we consider four proteins whose PAI values are 0.7, 0.1, 1.0 and 0.5. Left panel: areas between curves depict relative abundances computed using GemPAI as a function of the exponent base. Right panel: curves correspond to selected ratios of these abundances.

Here I consider a generalized exponentially modified PAI (GemPAI), which depends on a parameter corresponding to the base of the exponential function of PAI. GemPAI is given by the following formula:
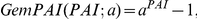
Obviously, emPAI = GemPAI(*,10). Figure 1 illustrates how the inferred relative abundances and their ratios depend on the base *a* of the exponential function for proteins with different values of PAI.

To determine for which value of the exponent base *a* the inferred abundances are most similar to the actual ones, I follow [1], and I use the same set of measured concentrations of 46 proteins from the whole lysate of mouse cells. I also use the same method of validating the approach by computing the “deviation factor”, D = exp(abs(log(*pc*/emPAI))), for each protein, where *pc* denotes the independently measured concentration of the protein (linearly scaled to minimize the average deviation factor over all detected proteins). This index is based on the ratio of the measured protein abundance to the abundance estimated using emPAI. Since it is based on ratios rather than differences, the deviation factor is less sensitive to outliers, and therefore suited to data with highly skewed marginal distributions (such as protein concentrations in the linear scale).

**Figure 2:**
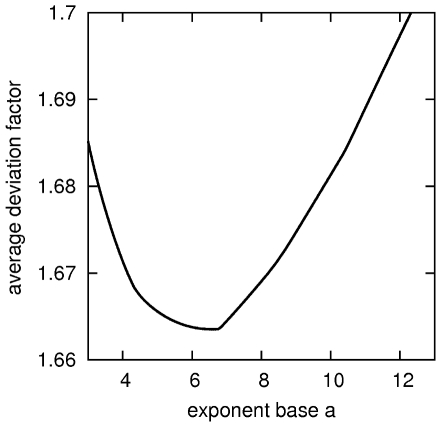
Optimization of the exponent base *a*. The average deviation factor <D> = 1/46 * ∑_i = 1,46_ exp(abs(log(*pc*_i_/GemPAI(PAI_i_;a)))) as a function of the exponent base *a* (for every *a*, *pc* is scaled to minimize <D>). The result is based on 46 proteins, identified and measured by [1].

**Figure 3:**
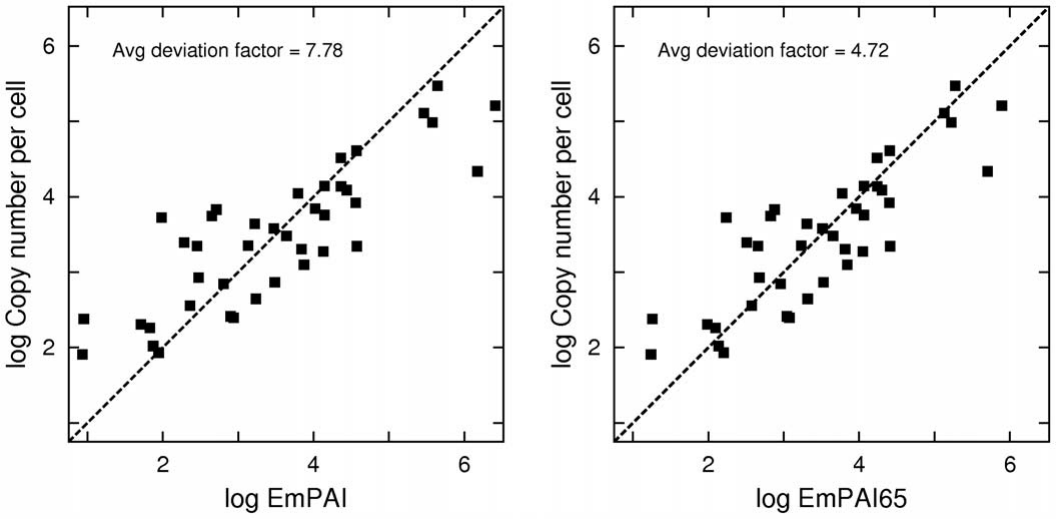
EmPAI and EmPAI65 applied to the E. coli data of [**7**] and [**8**]. The concentrations of 46 *E. coli* proteins measured by [7] and [8], normalized and plotted against emPAI (left panel) and against emPAI65 (right panel). In the log-log scale, proportionality corresponds to lines at a 45-degree angle, shown as the dashed line in each plot.

Here, I compute the generalized emPAI for the same 46 proteins for a wide range of exponent bases, from *a* = 3 to *a* = 15 with a step of 0.01. For each base, I estimate the best scaling factor to convert the relative abundances inferred from GemPAI into absolute concentrations, and next I calculate the deviation factors for all proteins. The average deviation factor as a function of the base is shown in Fig. 2. These results show that the average deviation factor is the lowest (corresponding to the best estimate of protein abundance by the generalized emPAI) for *a* = 6.50. Throughout this paper, I will denote GemPAI(*, 6.5) as emPAI65. EmPAI65 can be directly computed from PAI, or from emPAI using the following relation:
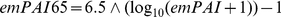
(1)To independently demonstrate the superiority of emPAI65 over emPAI, I have computed the values of emPAI65 for the proteins identified in the large-scale proteome profiling experiment of [7], and related them to the protein concentrations in *E. coli* cells measured by [8], using 42 data points analogously to the comparison presented in Fig. 2 of [7]. This dataset has a very high dynamic range, with the measured protein abundances spanning four orders of magnitude. I have computed the deviation factors for both emPAI and emPAI65 for the proteins plotted in Fig. 2 of [7]. The average deviation factor is 4.72 for emPAI65 and 7.78 for emPAI, again significantly lower for quantitation using base 6.5 rather than base 10. The measured protein concentrations are plotted against estimates with emPAI and emPAI65 in Figure 3, showing the greater deviation from proportionality in case of the standard emPAI. Note that unlike the mouse lysate data of [1], the *E. coli* data are derived from experiments by two research groups and a larger variance is expected, which is reflected by a higher average deviation factor. For this reason I did not use this dataset in the initial determination of the optimal exponent base.

**Figure 4:**
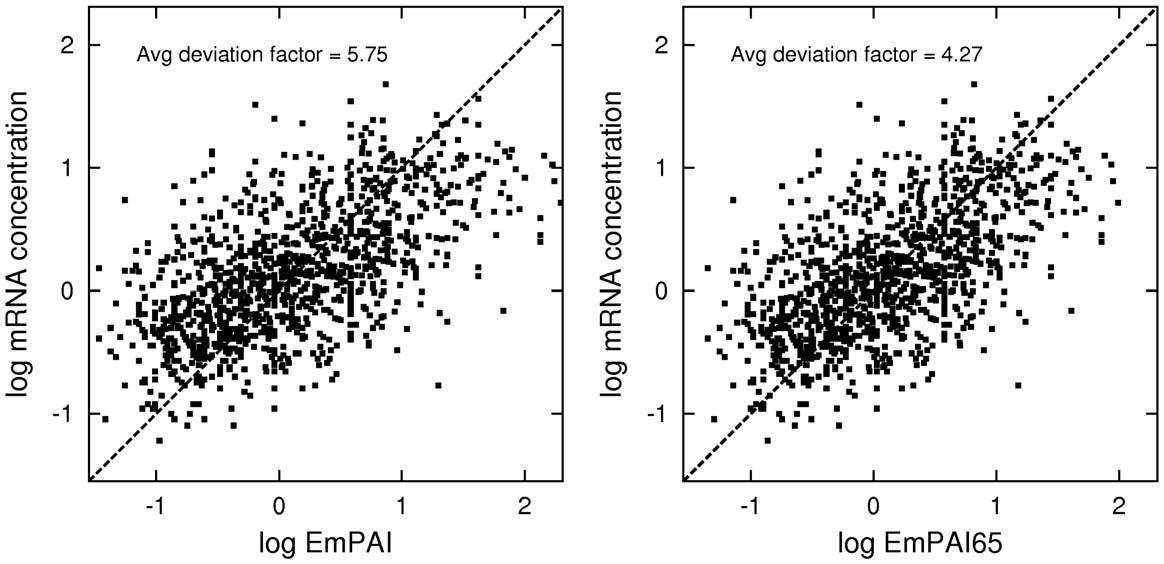
EmPAI and EmPAI65 applied to the E. coli data of [**10**]. The mRNA concentrations of 1270 *E. coli* proteins measured by [10], normalized and plotted against emPAI (left panel) and against emPAI65 (right panel). In the log-log scale, proportionality corresponds to lines at a 45-degree angle, shown as the dashed line in each plot.

Additional supporting evidence pointing to the improved performance of emPAI65 comes from comparing the inferred protein abundances with gene expression levels. Protein concentrations depend on mRNA abundances through translation, and although they are not exactly proportional to one another, they are expected to be significantly correlated, see e.g. [3], [9]. Comparing the correlation between mRNA concentrations and emPAI against the correlation between mRNA concentrations and emPAI65 may provide secondary evidence of the quality of either method of quantitation (of course, these correlations need to be computed in the linear scale). As an example, I have analyzed the data of [10], who report both protein identification results and a DNA microarray study for 1270 proteins in the membrane proteome of *Escherichia coli*. I find that in this experiment the Pearson correlation coefficient (in linear scale) of mRNA vs emPAI is 0.14, while the Pearson correlation coefficient of mRNA vs emPAI65 is 0.18. Additionally, I have compared the average deviation factors (as defined in [1]) between mRNA concentration and both versions of exponentially modified PAI. I find that the average deviation factor for emPAI, min*_s10_*(avg(exp(abs(log(s_10_*mRNA/*emPAI*))))), is 5.75, while its value for the proposed emPAI65, min*_s65_*(avg(exp(abs(log(s_65_*mRNA/*emPAI65*))))), is smaller and equals 4.27, which points to the relation between mRNA and emPAI65 being closer to linear than the relation between mRNA and emPAI (see also Figure 4). Again, both results strongly suggest a greater biological relevance of emPAI65 compared to emPAI.

A consequence of the difference between emPAI and emPAI65 is that the two methods produce different ratios of inferred protein abundances. To demonstrate the biological significance of this difference, I have computed the relative concentrations of all pairs of proteins inferred from PAI using both methods for the published data sets. I find that for many pairs of proteins the inferred abundance ratios are changed considerably. Specifically, in the data of Masuda et al [10] the ratio change is at least two-fold for 3% of the pairs, and 1.5-fold for 13% of the protein pairs. In the data of Ishihama et al [7], 18% of pairs exhibit a 2-fold change in inferred abundance ratios, while 30% pairs exhibit a 1.5-fold change. In conclusion - while the significance strongly depends on the experiment itself, specifically on the dynamic ratios of measured PAIs - the magnitude of the error introduced by using the standard EmPAI instead of emPAI65 may be substantial and should not be assumed to be negligible.

## Discussion

The relationship between peptide counts and protein concentration depends on a diverse spectrum of biochemical and instrumental phenomena. The complexity of the probability distributions describing them makes it very difficult to derive a theoretical formula for estimating relative abundances of proteins. Instead, empirical approximations are being used. While an infinitely broad range of mathematical functions may be proposed to estimate protein concentrations based on the numbers of observed peptides, Ishihama et al. have shown the near linear relation between the logarithm of protein abundance and the PAI, which supports quantitation based on an exponential function of PAI. I have analyzed the family of exponential functions parametrized by the base of the exponent, *a*. Using the same high-quality data as [1] and optimizing *a* by exhaustive 1-D grid search I conclude that the quantitation procedure performs best for *a* = 6.5. I define emPAI65 = 6.5∧PAI-1, and demonstrate that it performs better than standard emPAI for several other datasets. EmPAI65 is an empirically-derived formula and it is possible that a slightly different value of *a* could be derived when new high-quality data become available, however it is expected that the value will remain much closer to 6.5 than to 10. I therefore postulate to use and report emPAI65 rather than the original emPAI when estimating protein abundances from the numbers of observed peptides. Whereas the standard emPAI is computed by some of the existing software, it can be converted to emPAI65 with very simple arithmetics (Eq. 1), significantly improving the results.
